# An Uncommon Culprit: Cytomegalovirus Colitis Mimicking Malignancy in an Immunocompetent Patient

**DOI:** 10.7759/cureus.83290

**Published:** 2025-05-01

**Authors:** Zaraq R Khan, Rhiannon Huffines, Imad Majeed, Kenneth Hannan

**Affiliations:** 1 Infectious Diseases, University of Louisville Hospital, Louisville, USA; 2 Internal Medicine, University of Louisville Hospital, Louisville, USA; 3 Medicine, Khyber Teaching Hospital, Khyber Medical College, Peshawar, PAK

**Keywords:** cytomegalovirus (cmv), ganciclovir, immunosenescence, infectious colitis, rectal malignancy

## Abstract

Cytomegalovirus (CMV) can cause ulcerative lesions in the gastrointestinal tract, but it does not typically cause severe infections in immunocompetent patients. CMV colitis presenting as a rectal mass is a very rare manifestation often mimicking malignancy. This is an 89-year-old Caucasian female patient with a circumferential ulcerating mass and a sigmoid polyp that was found to be positive for CMV in the immunohistochemical staining. This is a rare presentation of CMV colitis, especially in an immunocompetent patient. The rarity of this disease presentation exemplifies the importance of a thorough workup for diagnosis.

## Introduction

Cytomegalovirus (CMV) belongs to the herpesvirus family and can cause an array of clinical manifestations ranging from asymptomatic infection in immunocompetent populations to life-threatening infections in immunocompromised populations [1]. Because the majority of the population gets an asymptomatic infection, it isn't easy to get accurate estimates of the prevalence, but according to one survey, seroconversion for CMV ranges from 40% in developed countries to 100% in some developing countries [2]. CMV is known to cause ulcerative lesions in the gastrointestinal tract irrespective of the immune status of the patient [3]. To the best of our knowledge, it is very rare for CMV infection to present as a rectal mass, and we have only a few case reports on it in the literature review [4, 5]. We present a case of CMV colitis in an 89-year-old female patient, which appeared as a rectal mass on colonoscopy.

## Case presentation

An 89-year-old Caucasian female patient with an unknown past medical history was brought to the hospital when she was found down in the toilet for more than 24 hours. As per the patient's son, who provided collateral history, she had diarrhea before admission. As the patient was not living with her son, he was unable to elaborate further on the symptoms of diarrhea.

Upon arrival at the hospital ED, the patient was found to be confused. She was mumbling words without any true meaning to them. As per the ED assessment, she met three out of four criteria for systemic inflammatory response syndrome (SIRS) with a white blood cell count of 13.7 × 10³/μL, a peripheral pulse rate of 106 beats per minute, and a respiratory rate of 26 breaths per minute. Her temperature was 97.8°F, and her blood pressure was 120/56 mmHg. She was also found to have acute kidney injury with a serum creatinine of 4.63 mg/dl (patient's baseline values: 0.9 - 1.1 mg/dl) (Table 1). Blood cultures were drawn before initiating empiric antibiotics, which remained negative at five days of incubation. The patient had a urine analysis that showed 50+ white cells/HPF, large leukocyte esterase, positive nitrite, and 4+ bacteria with reflex culture showing > 100,000 CFU/ml of *Escherichia coli.* The patient was initially started empirically on cefepime 1 gm q24 hours and vancomycin 1250 mg q24 hours after a loading dose of 1500 mg. The patient also had an MRI of the brain without contrast, which was unremarkable for any acute changes.

**Table 1:** The patient's key blood-based laboratory findings during hospitalization

Laboratory test	Patient value	Reference range
White clood cell count	13.7 × 10³/μL	4.0 – 11.0 × 10³/μL
Serum creatinine	4.63 mg/dL	0.6 – 1.3 mg/dL
Hemoglobin (Day 4)	13.5 g/dL	12.0 – 16.0 g/dL (female)
Hemoglobin (Day 6)	12.1 g/dL	12.0 – 16.0 g/dL (female)

On hospital day 4, the patient developed hematochezia. As per the patient's nurse, she had three episodes of solid bowel movements covered in blood. The gastroenterology team was taken on board, and it was decided to perform an inpatient colonoscopy due to the patient's gradual downtrend of hemoglobin from 13.5 g/dl on hospital day 4 to 12.1 g/dl on hospital day 6. The patient's *Escherichia coli* in urine was later found to be susceptible to ampicillin/sulbactam, so her antibiotics were switched to ampicillin/sulbactam 2 gm/1 gm every 6 (q6) hours on hospital day 6. Her stool specimen was sent for an enteric pathogen panel (polymerase chain reaction (PCR)-based test), which was tested for *Campylobacter*, *Salmonella*, *Shigella*, *Vibrio*, *Yersinia*, rotavirus A, Shiga toxin 1, and Shiga toxin 2, but came back negative. She also tested negative for *Clostridium difficile* toxin antigen and gene PCR.

**Figure 1:**
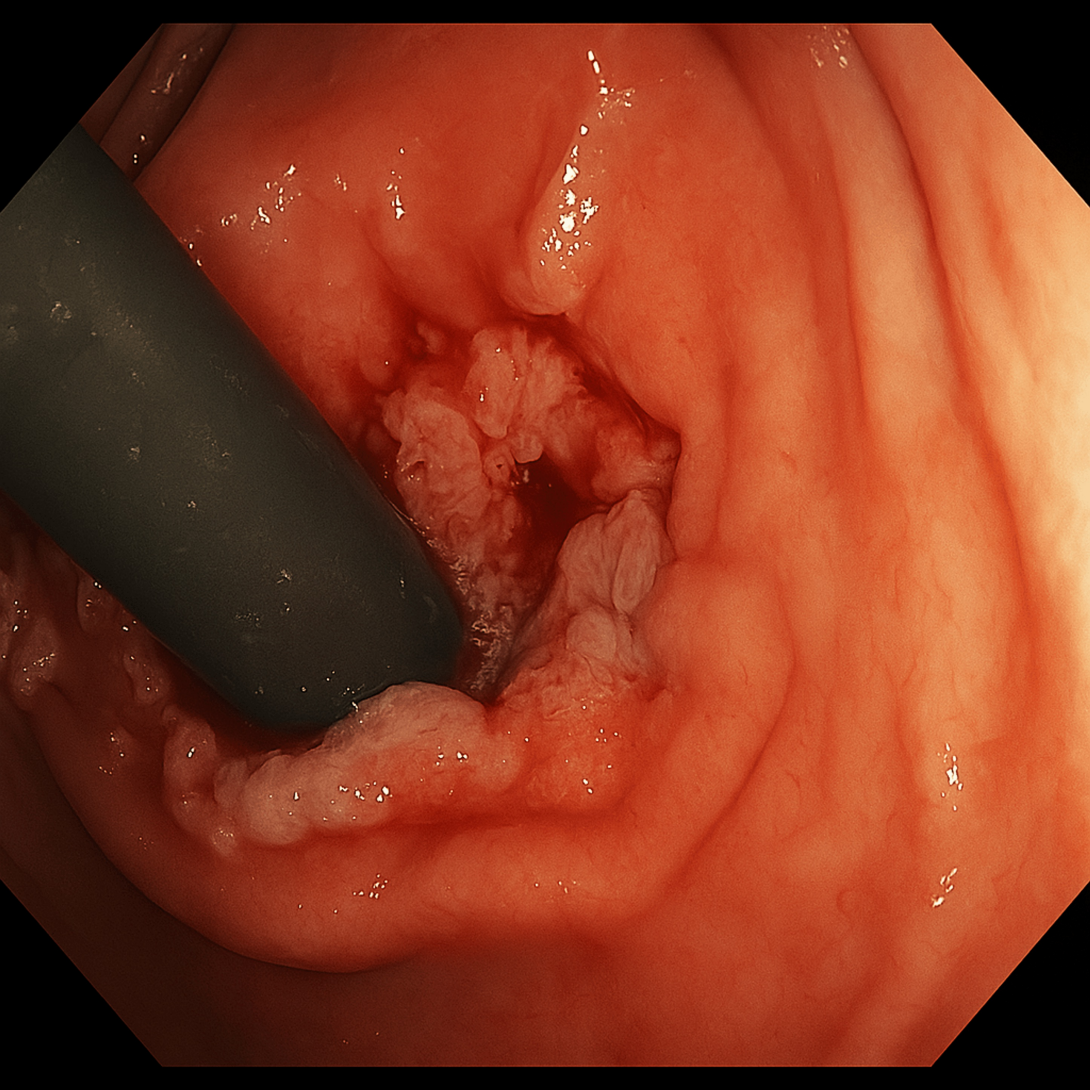
Circumferential rectal mass on colonoscopy

**Figure 2:**
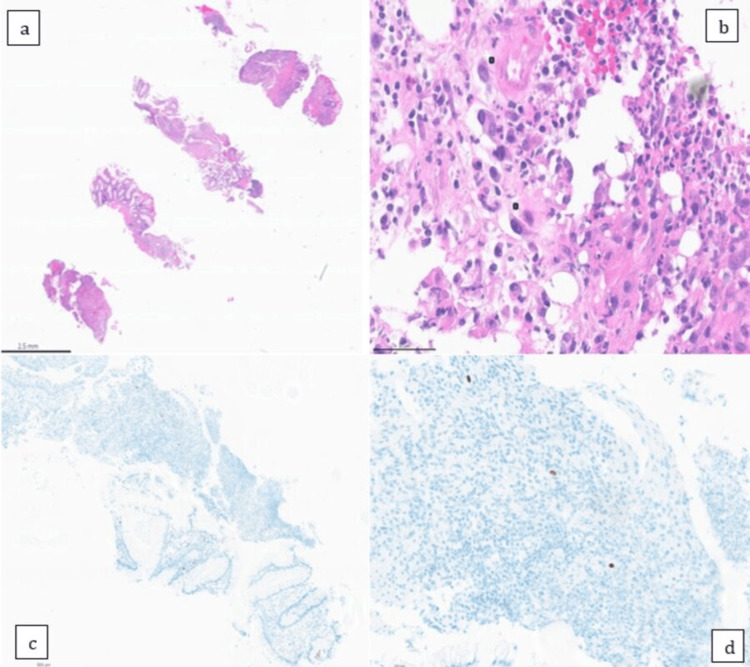
A: H&E-stained section with 1x magnification. b: H&E-stained section with 40x magnification. c: immunohistochemical stain for CMV with 5x magnification. d: immunohistochemical stain for CMV with 20X magnification H&E: hematoxylin and eosin; CMV: cytomegalovirus

The patient was found to have a circumferential ulcerating mass and a sigmoid polyp on colonoscopy (Figure 1). Biopsies from the mass in the rectum and the polyp were sent for pathology. The pathology report from the polyp was consistent for tubular adenoma, while that for the rectal mass showed squamocolumnar junctional mucosa with ulceration, inflamed granulation tissue response, and atypia consistent with reactive atypia. The rectal mass was found to be negative for any evidence of malignancy, but the immunohistochemical stain was positive for CMV, as shown in Figure 2. The serum CMV DNA quantitative PCR was < 35 IU/ml. The patient was started on intravenous ganciclovir 150 mg every 12 hours based on an adjusted body weight of 52 kg. To complete the workup for any underlying immunodeficiency, the patient was tested for CD4 count, which was 811 cells/microliter (47.7%). Her HIV screening test was negative, and serum immunoglobulins were within the normal range.

The patient's hematochezia improved progressively on day 3 of the treatment. She was treated for a total of two weeks before she was eventually transferred to a sub-acute rehabilitation center. The plan was to perform an outpatient sigmoidoscopy to evaluate for resolution of the mass, but unfortunately, the patient was lost to follow-up.

## Discussion

CMV colitis is often asymptomatic in immunocompetent patients. Although it can present with symptoms of fever, diarrhea, hematochezia, and abdominal pain, it is often self-limited and may not require treatment with antivirals [6,7]. Known risk factors for CMV infection in healthy patients include older age, kidney disease, hemodialysis, red blood cell transfusions, use of antibiotics, use of corticosteroids, or exposure to intensive care units [8]. CMV infections are more common in immunocompromised patients, especially patients infected with HIV or patients with solid organ transplants. In this population, CMV can cause severe manifestations, including colon perforation, toxic megacolon, pseudomembrane formation, ischemic colitis, and even severe hemorrhage [6]. CMV is also found in patients with inflammatory bowel disease, including Crohn’s disease and ulcerative colitis [9]. Our patient had hematochezia but no evidence of any abdominal perforation. Old age was the only risk factor in our patient. Workup done to determine the patient’s immunocompetency status, including CD4 counts, serum immunoglobulins, and HIV, was all negative.

There are multiple modalities to diagnose CMV infections. Immune serology is one method of diagnosis of CMV infection, but has its drawbacks, as IgM titers rise initially with infection but may be persistently positive for months. Similarly, IgG may take several weeks to rise [6]. Immunoserology is therefore not a diagnostic tool for CMV colitis [6]. Most adults who had previous exposure to CMV will have positive test results irrespective of active CMV colitis [10]. Endoscopic findings of CMV colitis are often nonspecific. They can include several well-defined ulcers with a punched-out appearance [10, 11]. There are only two case reports of CMV colitis presenting as an inflammatory tumor-like mass in an immunocompetent patient [12, 13]. In these instances where diagnosis may be uncertain, histological examination of tissue specimens taken from the edge of the ulcers can provide clarity. Histology of colon biopsies stained with hematoxylin and eosin (H&E) can reveal “owl eye inclusion bodies,” which are very typical and specific for CMV. H&E has lower sensitivity compared to immunohistochemistry [6]. The gold standard of CMV diagnosis is CMV-specific immunohistochemistry, which labels CMV antigen in infected cells [14]. PCR tests can quantitatively detect viral DNA. Though not as specific as immunohistochemistry for the diagnosis of CMV, PCR can be a helpful adjunct to other methods of diagnosis by quantifying viral load burden [14]. PCR is more specific to CMV colitis when performed on samples from colon biopsies themselves [10]. In our patient, a colonoscopy was performed, and a circumferential ulcerating mass was found. Biopsy performed during colonoscopy was positive for CMV on the immunohistochemistry stain, which is the gold standard of diagnosis. The mass biopsy was also found to have no evidence of malignancy. The serum CMV DNA quantitative PCR was < 35 IU/ml. Other infectious work-ups, including *Campylobacter*, *Salmonella*, *Shigella*, *Vibrio*, *Yersinia*, rotavirus A, Shiga toxin 1, Shiga toxin 2, and *Clostridioides difficile* toxin, were all negative.

The first-line drug for treating symptomatic CMV colitis is ganciclovir, which can be prepared orally or intravenously [10]. The duration of treatment is usually two to three weeks and typically can be transitioned from IV to oral after three to five days [10]. Ganciclovir can be associated with negative side effects, including myelosuppression, hepatotoxicity, nephrotoxicity, or central nervous system disturbance. [10]. If a patient has evidence of resistance or intolerance to ganciclovir, other options may include foscarnet or cidofovir [15].

It is debated whether immunocompetent individuals should be treated with antivirals, given the side effects that may occur. In practice, it is generally agreed upon to treat CMV colitis with severe presentations even in immunocompetent patients.

Given the patient’s severe presentation and evidence of CMV infection, we chose to treat the patient with intravenous ganciclovir 150 mg q12 hours, based on an adjusted body weight of 52 kg. The patient was treated for two weeks with improvement of hematochezia. The plan was to perform an outpatient sigmoidoscopy to evaluate for resolution of the mass, but unfortunately, the patient was lost to follow-up.

## Conclusions

CMV colitis is usually asymptomatic in immunocompetent patients but can nonetheless be a cause of severe morbidity in certain patient populations. This was a rare presentation in an immunocompetent patient with no obvious risk factors. We believe that cases like these will help the medical fraternity know more about the rare manifestations of CMV infections and pave the way for more research work into the duration of treatment for CMV infections in immunocompetent populations.
